# Mitochondrial insights: key biomarkers and potential treatments for diabetic nephropathy and sarcopenia

**DOI:** 10.3389/fcell.2025.1596204

**Published:** 2025-07-09

**Authors:** Yi Wei Chen, Shan He, Yu Wang, Lian Ying Hu, Qin Kai Chen, Si Yi Liu

**Affiliations:** ^1^ Department of Orthopedics, Jiujiang University Affiliated Hospital, Jiujiang, China; ^2^ Department of Nephrology, The First Affiliated Hospital, Jiangxi Medical College, Nanchang University, Nanchang, China

**Keywords:** diabetic nephropathy, sarcopenia, machine learning, mitochondria-related genes, molecular docking

## Abstract

**Introduction:**

Clinical studies reveal bidirectional links between sarcopenia (SP) and diabetic nephropathy (DN). Damage to mitochondria in DN may result in diminished energy production, which consequently triggers SP. Consequently, mitochondria seem to function as critical nodes connecting DN with SP. The objective of this research was to pinpoint biomarkers associated with mitochondrial dysfunction in DN and SP.

**Methods:**

By analyzing the Gene Expression Omnibus (GEO) repository, we identified shared differentially expressed genes (DEGs) in the DN (GSE96804, GSE30528) and SP (GSE1428, GSE136344) datasets that displayed similar expression trends in mitochondrial genes. Using Least Absolute Shrinkage and Selection Operator (LASSO), Support Vector Machine (SVM), Extreme Gradient Boosting (XGB), and Random Forest (RF) algorithms, we identified three key mitochondrial hub genes. Diagnostic nomograms were created to predict DN and SP risk. We assessed immune infiltration using CIBERSORT and built a drug-gene network with Cytoscape. Molecular docking determined binding affinities between potential drugs and hub genes, which were validated in the datasets.

**Results:**

Analysis of GEO datasets identified 80 shared DEGs between DN and SP, including 10 mitochondria-related genes. Utilizing four machine learning algorithms (LASSO, SVM, XGBoost, RF), we pinpointed three mitochondrial hub genes. Subsequent validation confirmed two key mitochondria-related genes - *HTT* and *TTC19* - as shared diagnostic biomarkers for both DN and SP. These biomarkers demonstrated strong diagnostic power (AUC >0.8), leading to the construction of diagnostic nomograms. Immune infiltration analysis revealed elevated M1 macrophages in DN and increased M2 macrophages in SP, with both biomarkers showing significant correlations with various immune cells. Gene set enrichment analysis linked *HTT* and *TTC19* to mitochondrial metabolic processes. Crucially, *in silico* drug prediction identified 156 potential drugs, and molecular docking confirmed a high binding affinity between acetaminophen and the *HTT* protein.

**Conclusion:**

Our study identifies *HTT* and *TTC19* as novel mitochondria-immune related biomarkers common to both DN and SP, providing insights into their shared pathogenesis involving mitochondrial dysfunction and immune dysregulation. The computational prediction of acetaminophen interaction highlights a potential avenue for therapeutic exploration. Further clinical and mechanistic studies are warranted to validate these findings and elucidate the underlying pathways.

## 1 Introduction

Diabetic nephropathy (DN) represents the predominant primary etiology of end-stage renal disease (ESRD) (Yu and Bonventre, 2018). Research indicates that approximately 30%–40% of individuals with Type 1 Diabetes Mellitus (T1DM) will develop DN, with around 50% of these cases advancing to ESRD (Kato and Natarajan, 2019). The pathogenesis of DN is characterized by a complex interaction of metabolic and inflammatory responses, hemodynamic factors, and various etiological determinants (Kato and Natarajan, 2019). Furthermore, oxidative stress, mitochondrial dysfunction, and renal hypoxia are implicated in the progression and exacerbation of DN. Mitochondrial dysfunction has emerged as a critical factor in the progression of DN, given its essential role in cellular energy metabolism and the regulation of reactive oxygen species (ROS). Studies have demonstrated that elevated blood glucose levels result in the overloading of the electron transport chain, subsequently increasing the production of ROS. This increase in ROS induces DNA damage and reduces the activity of glyceraldehyde-3-phosphate dehydrogenase (GAPDH) (Takasu et al., 2025). Additionally, the reduction of superoxide within the mitochondria exacerbates the inflammatory response by impairing vascular function and activating the nuclear factor-κB (NF-κB) pathway in B cells (Takasu et al., 2025; Wei and Szeto, 2019). The consequent impairment of mitochondrial function ultimately initiates apoptosis and cell death, contributing to the onset and progression of DN (Chang et al., 2021). Sarcopenia (SP) is a skeletal muscle disorder marked by the gradual decline in muscle mass and strength. The adverse effects of SP primarily encompass an elevated risk of falls, fractures, and disability, potentially resulting in the loss of independent living capabilities among older adults. Additionally, SP is associated with heightened rates of all-cause mortality and disability in this population (Cruz-Jentoft and Sayer, 2019). In comparison to youthful musculature, aging muscles exhibit an increased production of ROS and a concomitant decline in both oxygen consumption and ATP synthesis (Kamarulzaman and Makpol, 2025). In senescent skeletal muscles, various stimuli, including calcium, oxidative stress, and TNF-α, have the potential to induce apoptosis (Bottoni et al., 2019). Furthermore, alterations in mitochondrial protein interactions, cristae architecture, and mitochondrial networks may result in ADP insensitivity, thereby contributing to the development of SP [7].

The association between DN and SP is receiving increasing scholarly attention. A meta-analysis has identified DN as a risk factor for the onset of SP in individuals with diabetes (Feng et al., 2022). Additionally, emerging studies have underscored SP as a distinct risk factor for the development of DN. Furthermore, recent research has highlighted SP as an independent risk factor for DN (Huang et al., 2022). Despite the prevalence of SP and DN as comorbidities in elderly diabetic patients, no studies to date have confirmed a shared pathogenic mechanism underlying these two conditions. This underscores the necessity of investigating their common molecular pathways. In patients with DN, elevated serum levels of oxidative stress markers have been observed, which are significant contributors to renal tubular cell damage and the progression of DN (Xu et al., 2016). Mitochondria, serving as the cellular energy centers, are vital for maintaining cellular homeostasis but are particularly vulnerable to oxidative stress-induced damage (Min et al., 1985). Mitochondrial damage is pivotal in the pathophysiology of DN and SP, potentially acting as a nexus between these conditions. Under hyperglycemic conditions, mitochondria generate an excessive amount of ROS, which induces oxidative stress and results in damage to glomerular and tubular cells, as well as mitochondrial dysfunction (Takasu et al., 2025). This dysfunction not only impairs the energy metabolism of renal cells, thereby exacerbating renal damage (Takasu et al., 2025), but may also activate apoptotic signaling pathways, leading to the apoptosis of muscle cells and consequently facilitating the progression of SP (Bottoni et al., 2019). Thus, the identification of mitochondrial-related biomarkers common to both DN and SP could provide valuable insights for the diagnosis and treatment of these conditions.

Our research endeavor seeks to synthesize various public datasets to identify mitochondrial genes linked to DN and SP, and to assess their diagnostic significance in these conditions. Through the application of differential expression analysis, machine learning algorithms, and immune infiltration analysis, we aim to elucidate the potential molecular mechanisms underlying these diseases. Our findings reveal druggable targets and diagnostic markers associated with the shared pathological mechanisms between DN and SP.

## 2 Materials and methods

### 2.1 Data download and integration

The datasets for DN, GSE96804 (Pan et al., 2018; Shi et al., 2018) and GSE30528 (Woroniecka et al., 2011), and the datasets for sarcopenia (SP), GSE1428 (Giresi et al., 2005) and GSE136344 (Gueugneau et al., 2021), were all retrieved from the GEO database (https://www.ncbi.nlm.nih.gov/geo/). Four datasets were analyzed using distinct microarray platforms: GSE30528 (GPL571) and GSE1428 (GPL96) employed Human Genome U133A arrays (v2.0 and original respectively), whereas GSE96804 (GPL17586) and GSE136344 (GPL5175) utilized Human Transcriptome Array 2.0 and Exon 1.0 ST Array platforms correspondingly. In the DN datasets, two independent DN cohorts were analyzed: GSE30528 (13 controls vs. 9 DN cases) and GSE96804, which comprised renal tubular specimens from 20 healthy donors and 41 DN patients. The subsequent integration of these datasets resulted in a combined total of 33 control and 50 DN samples, ensuring statistical robustness for the differential expression analysis. In the SP datasets, GSE1428 included 10 young samples and 12 elderly samples, and GSE136344 included 11 young samples and 12 elderly samples. The data from the two datasets were integrated, resulting in a combined dataset with 21 young and 24 elderly normal tissue samples. Specific dataset information is provided in Table 1. We used the R package “sva” (Leek et al., 2012) to perform batch effect removal on the DN datasets GSE30528 and GSE96804, and the SP datasets GSE1428 and GSE136344, respectively, to obtain the integrated datasets DN-Datasets and SP-Datasets. The study workflow is illustrated in Supplementary Figure S1.

**TABLE 1:** GEO dataset information list.

	GSE30528	GSE96804	GSE1428	GSE136344
Platform	GPL571	GPL17586	GPL196	GPL5175
Experiment type	Expression profiling by array	Expression profiling by array	Expression profiling by array	Expression profiling by array
Species	*Homo sapiens*	*Homo sapiens*	*Homo sapiens*	*Homo sapiens*
Tissue	Glomeruli	Glomeruli	Vastus lateralis muscle	Vastus lateralis muscle
Samples in control group	Control (13)	Control (20)	10 young (19–25 years old)	11 young samples
Samples in disease group	DN (9)	DN (41)	12 older (70–80 years old)	12 elderly samples
Reference	Transcriptome analysis of human diabetic kidney disease	Dissection of Glomerular Transcriptional Profile in Patients With Diabetic Nephropathy: SRGAP2a Protects Podocyte Structure and Function	Identification of a molecular signature of sarcopenia	Muscle Proteomic and Transcriptomic Profiling of Healthy Aging and Metabolic Syndrome in Men

DN, diabetic nephropathy.

### 2.2 Differential expression gene analysis

We used the “limma” package in R software to perform differential analysis on the expression profile data of the DN - Datasets and SP - Datasets respectively, and screened the genes with the criteria of |logFC| > 0.25 and p. value <0.05. The differential expression analysis outcomes were visualized through a volcano plot, which was created utilizing the R package ggplot2.

### 2.3 Identification of genes with the same trend and mitochondria-related DEGs

We screened the differentially expressed genes (DEGs) that showed a common expression trend. By searching the GeneCard (Stelzer et al., 2016) database (https://www.genecards.org/), we identified 2,676 mitochondria-related genes using a correlation score exceeding 1 as the filtering criterion. The overlap between genes co-expressed differentially and those associated with mitochondria was utilized to pinpoint differentially expressed genes linked to mitochondrial functions.

### 2.4 Gene function enrichment analysis

The “enrichplot” package was employed to visualize the Gene Ontology (GO) (Gene Ontology Consortium, 2015) enrichment outcomes, encompassing biological processes, cellular components, and molecular functions. Furthermore, a network diagram was created to illustrate the enrichment findings from the Kyoto Encyclopedia of Genes and Genomes (KEGG) (Kanehisa and Goto, 2000) analysis.

### 2.5 Selection of key mitochondrial genes using machine learning algorithms

Least Absolute Shrinkage and Selection Operator (LASSO) is used for initial dimensionality reduction and linear feature screening; Support Vector Machine (SVM) handles nonlinear classification of high-dimensional small-sample data; and Random Forest (RF) and Extreme Gradient Boosting (XGB) capture complex interaction effects among genes through integrated learning. Ultimately, only genes selected in all four algorithms are defined as key biomarkers to ensure the robustness and interpretability of the results. We employed the LASSO, XGB, SVM, and RF methods to identify critical prognostic genes, and we constructed regression models with the aid of the “glmnet” package in R. The penalty coefficient was established using a tenfold cross-validation process. Subsequently, the Random Forest model was created with the “randomForest” package in R, following which the top ten variables were selected based on their importance scores. The XGB model was developed with the assistance of the “xgboost” package in R, which facilitated the selection of key variables based on feature importance scores (Gain or Cover) through tenfold cross-validation. For SVM, the “e1071”package was utilized to perform recursive feature elimination (SVM-RFE) for variable selection. A Venn diagram was utilized to determine the overlapping DEGs related to mitochondria, which were identified by the LASSO, XGB, SVM, and RF methods.

### 2.6 Diagnostic model construction and evaluation

We applied the “rms” (Xu et al., 2021) package to construct a Nomogram model to predict the risk of patients. A calibration curve was used to determine the degree of match between our predicted values and the reality. Decision curve analysis (DCA) was performed to evaluate whether model - based decisions were beneficial to patients.

### 2.7 Immune infiltration analysis

We utilized the CIBERSORT tool, an algorithm for Cell Type Identification By Estimating Relative Subsets Of RNA Transcripts (Kawada et al., 2021), to deconvolute the cellular composition of intricate tissues from gene expression profiles (https://cibersort.stanford.edu/). We carried out an analysis to investigate immune cell infiltration in the samples from patients, comparing the disease group with the normal control group. The “e1071”and “preprocessor” packages in R software were used in combination with the CIBERSORT algorithm. Additionally, we assessed the correlation between hub DEGs associated with mitochondria and the aforementioned immune cells, and we have depicted these findings in a heat map format. Furthermore, we measured the immune cell composition within each individual sample. The simulation was conducted with a total of n calculations, and outcomes with a significance level of p < 0.05 were filtered and kept for analysis.

### 2.8 GSEA analysis

Utilizing the “enrichR” package within R, we performed Gene Set Enrichment Analysis (GSEA) (Subramanian et al., 2005). The hallmark gene set and immune signature gene set were downloaded as the background sets for the enrichment analysis. Subsequently, we computed the enrichment score and p-value for each gene set via the GSEA method.

### 2.9 Drug prediction and molecular docking

First, we used the Comparative Toxicogenomics Database (CTD) (Davis et al., 2021) to predict drugs for the genes (http://ctdbase.org/.) A miRNA - drug regulatory network was constructed and visualized using Cytoscape. Structural data for target proteins and drug components were obtained from the Protein Data Bank (PDB, http://www.rcsb.org) and the PubChem database (Cob-Calan et al., 2019) (https://pubchem.ncbi.nlm.nih.gov). Subsequently, the protein structures were transformed into *.pdbqt format, following which molecular docking was carried out using the web-based tool CB-dock2 (https://cadd.labshare.cn/cb - dock2). The folding patterns and molecular interactions between the target protein and ligand molecules were examined to obtain the binding energy for screening.

### 2.10 Statistical analysis

All data processing and analysis in this study were conducted using R software (Version 4.3.3). For the comparison of two groups of continuous variables, the independent Student’s t-test was employed to estimate the statistical significance of normally distributed variables, while the Mann-Whitney U test (also known as the Wilcoxon rank-sum test) was utilized to analyze differences between non-normally distributed variables. For comparisons involving three or more groups, the Kruskal–Wallis test was applied. Chi-square tests or Fisher’s exact tests were used to assess the statistical significance of categorical variables between two groups. Spearman’s correlation method was employed to compute the correlation coefficients between various molecules. Unless otherwise specified, all statistical P values were two-tailed, with a significance level set at P < 0.05.

## 3 Results

### 3.1 Differential analysis of datasets

**FIGURE 1:**
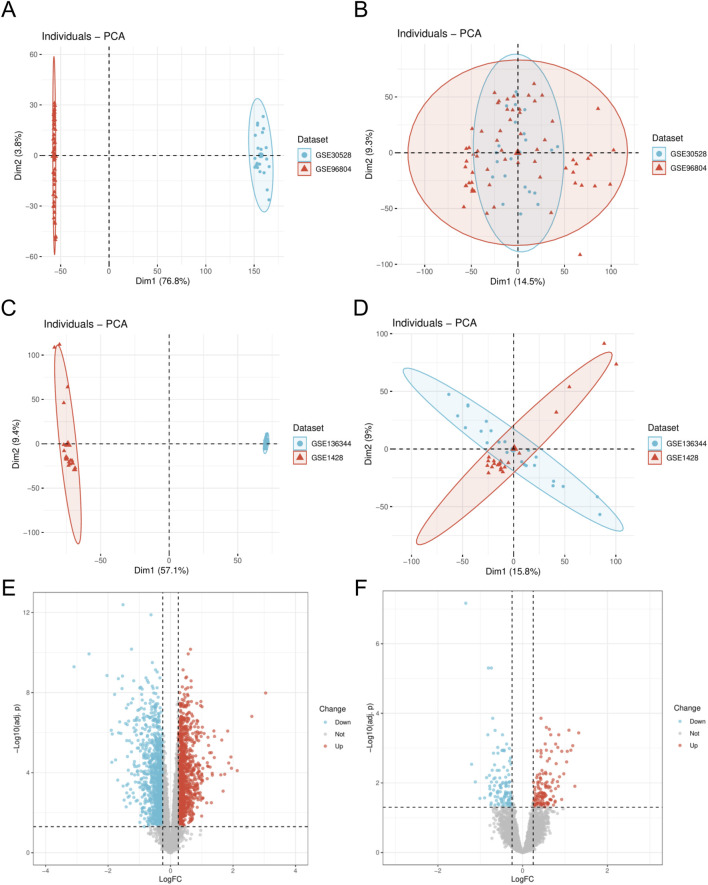
Dataset correction and analysis of differentially expressed genes. **(A)** PCA plot of DN- Datasets before correction. **(B)** PCA plot of the corrected DN-Datasets. **(C)** PCA plot of SP- Datasets before correction. **(D)** PCA plot of the corrected SP- Datasets. **(E)** Volcano plot of differential analysis results between DN and Control groups in the DN- Datasets dataset. **(F)** Volcano plot of differential analysis results between SP and Control groups in the SP- Datasets dataset. DN, Diabetic nephropathy; SP, Sarcopenia. The Diabetic nephropathy (DN) dataset GSE30528 is in light blue, and the Diabetic nephropathy (DN) dataset GSE96804 is in light red. The Sarcopenia (SP) dataset GSE136344 is in light blue, and the Sarcopenia (SP) dataset GSE1428 is in light red.

We initially employed the R package “sva” to perform batch effect removal on the DN-Dataset and SP-Datasets, resulting in the batch-corrected DN-Datasets and SP-Datasets. Subsequently, we compared the datasets before and after batch effect correction using Principal Component Analysis (PCA), as illustrated in Figures 1A–D. The PCA results indicate that the batch effects in the samples of the datasets have been largely eliminated following the batch removal procedure. Differential analysis was conducted using the “limma” package, and the results were visualized through a volcano plot. In the DN-Datasets, a total of 2,308 differentially expressed genes were identified, comprising 1,171 upregulated genes and 1,137 downregulated genes (Figure 1E). Furthermore, within the SP-Datasets, we detected 253 genes displaying differential expression, comprising 117 that were upregulated and 136 that were downregulated (Figure 1F).

### 3.2 Identification of mitochondrial-related genes

**FIGURE 2:**
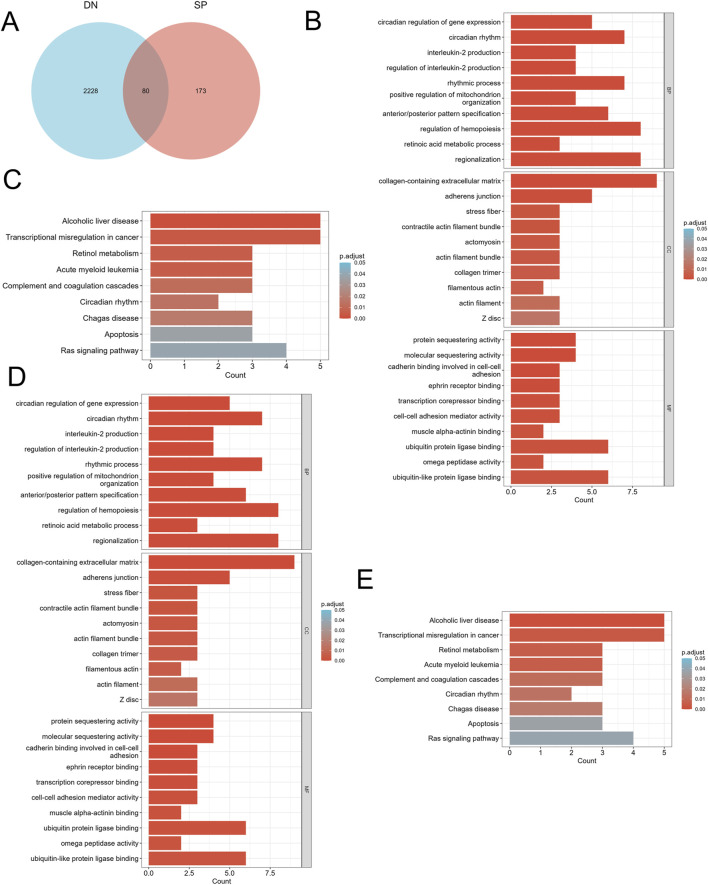
Identification of DEGs and functional enrichment between DN and SP. **(A)** Venn diagram of the DEGs. **(B)** GO enrichment analyses of DEGs with the same expression trends in DN; **(C)** KEGG enrichment analyses of DEGs with the same expression trends in DN. **(D)** GO enrichment analyses of DEGs with the same expression trends in SP. **(E)** KEGG enrichment analyses of mitochondria-related DEGs in SP. DN, Diabetic nephropathy; SP, Sarcopenia; DEGs, differentially expressed genes; GO, Gene ontology; KEGG, Kyoto encyclopedia of genes and genomes; BP, Biological process; CC, Cellular component; MF, Molecular function. The screening criteria for GO/KEGG enrichment items were p. Adj <0.05 and FDR value (q. value) <0.25, and the p value correction method was Benjamini–Hochberg (BH).

**FIGURE 3:**
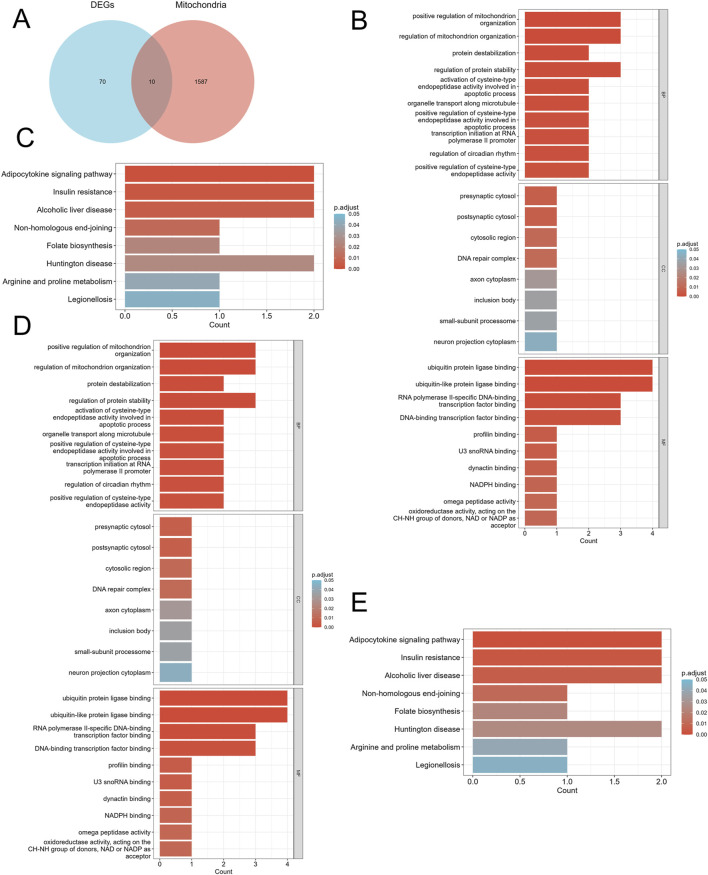
Identification of mitochondria-related DEGs and functional enrichment between DN and SP. **(A)** Venn diagram of the mitochondria-related DEGs. **(B)** GO enrichment analyses of mitochondria-related DEGs in DN; **(C)** KEGG enrichment analyses of mitochondria-related DEGs in DN. **(D)** GO enrichment analyses of mitochondria-related DEGs in SP; **(E)** KEGG enrichment analyses of DEGs with the same expression trends in SP. DN, Diabetic nephropathy; SP, Sarcopenia; DEGs, differentially expressed genes; GO, Gene ontology; KEGG, Kyoto encyclopedia of genes and genomes; BP, Biological process; CC, Cellular component; MF, Molecular function. The screening criteria for GO/KEGG enrichment items were p. Adj <0.05 and FDR value (q. value) <0.25.

We screened differentially expressed genes (DEGs) exhibiting similar expression trends (Figure 2A) and selected those that intersected with mitochondria-related genes (Figure 3A). This resulted in the identification of ten mitochondria-related DEGs: *CKB*, *FAM162A*, *HSPE1*, *HTT*, *NFKBIA*, *PPARGC1A*, *PRKDC*, *QDPR*, *TTC19*, and *UCHL1*. In the biological process (BP) enrichment analysis, DEGs with similar expression trends were primarily associated with the regulation of cellular circadian rhythms. This is closely linked to processes such as the cell cycle and metabolism (Figure 2B). During the analysis of cellular component (CC) enrichment, the main enrichment was observed in the extracellular matrix. In the molecular function (MF) enrichment analysis, the predominant enrichment was related to transcriptional corepressor binding (Figure 2C,D). KEGG pathway analysis indicated that these genes were enriched in pathways associated with circadian rhythms, apoptosis, and the RAS signaling pathway (Figure 2E). For the BP enrichment analysis of mitochondria-related DEGs, the primary enrichment was related to circadian rhythms. In the CC enrichment analysis, it was mainly associated with presynaptic and postsynaptic cytoplasm, and in the MF enrichment analysis, it was predominantly linked to transcription factor binding (Figures 3B–D). KEGG pathway analysis demonstrated that these genes were enriched in pathways related to insulin resistance, adipokine signaling, and arginine and proline metabolism (Figure 3E).

### 3.3 Identifying key mitochondrial genes and their diagnostic significance

**FIGURE 4:**
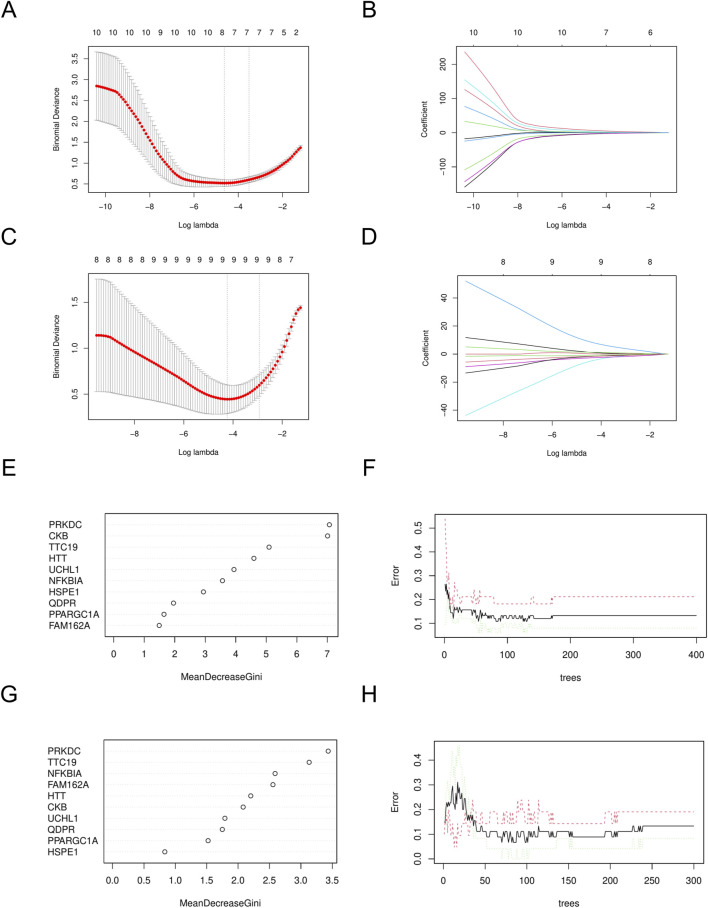
Construction of machine learning models. **(A)** Variables determined by LASSO analysis in DN- Datasets. **(B)** LASSO Coefficient distribution map-LASSO coefficient distribution of all variables in DN- Datasets. **(C)** variables determined by LASSO analysis in SP- Datasets. **(D)** LASSO Coefficient distribution map-LASSO coefficient distribution of all variables in SP- Datasets. **(E)** The relative importance of genes in random forest models in DN- Datasets. **(F)** Confidence intervals for error rates of random forest models in DN- Datasets. **(G)** The relative importance of genes in random forest models in SP- Datasets. **(H)** Confidence intervals for error rates of random forest models in SP- Datasets. DN, Diabetic nephropathy; SP, Sarcopenia. LASSO, Least Absolute Shrinkage and Selection Operator.

**FIGURE 5:**
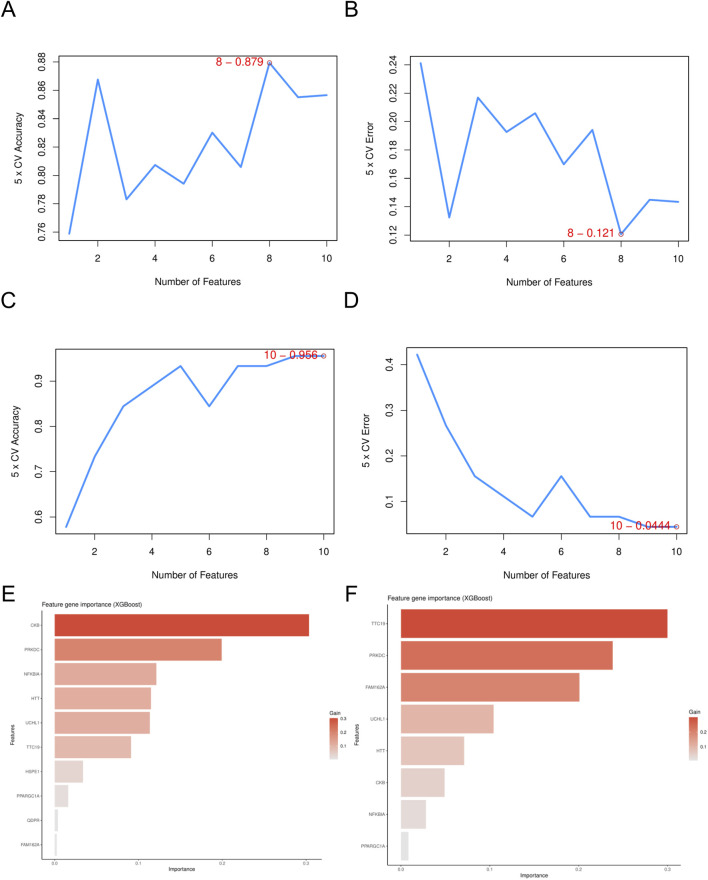
Screening of mitochondria-related hub genes. **(A)** The accuracy of the SVM model in DN- Datasets. **(B)** The accuracy of the SVM model in SP- Datasets. **(C)** The error of the SVM model in DN- Datasets. **(D)** The error of the SVM model in SP- Datasets. **(E)** The feature gene importance for the XGB model in DN- Datasets. **(F)** The feature gene importance for the XGB model in SP- Datasets. DN, Diabetic nephropathy; SP, Sarcopenia; SVM, Support Vector Machine; XGB, eXtreme Gradient Boosting.

**FIGURE 6:**
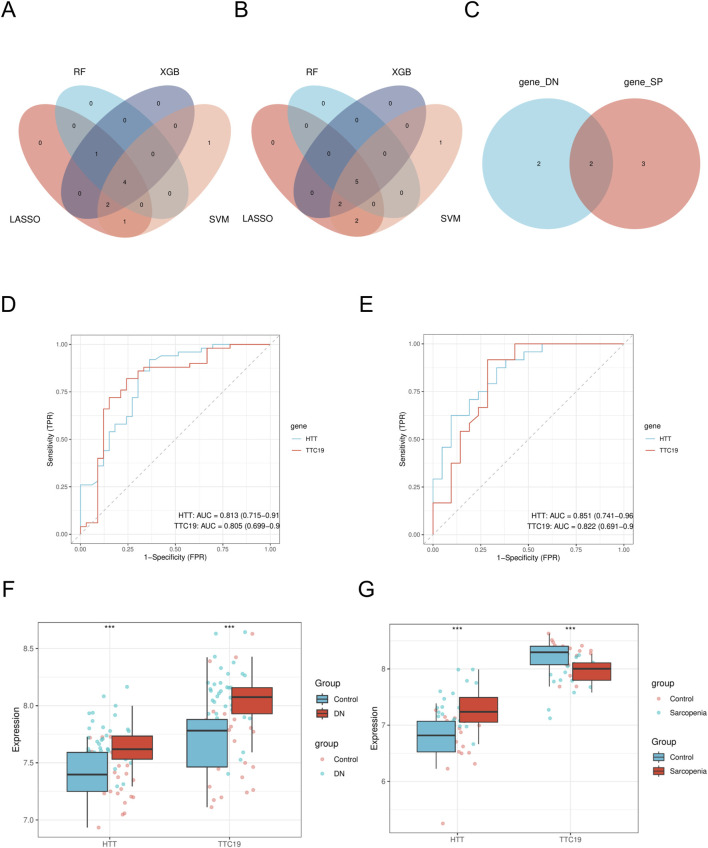
Identification of mitochondria-related hub genes. **(A)** The intersected genes of three machine learning analyses shown by Venn diagram in DN- Datasets. **(B)** The intersected genes of three machine learning analyses shown by Venn diagram in SP- Datasets. **(C)** Venn diagram of mitochondria-related hub genes. **(D)** ROC curve of *HTT* and *TTC19* between different groups (DN/Control) of DN-Datasets. **(E)** ROC curve of *HTT* and *TTC19* between different groups (SP/Control) of SP-Datasets. **(F)** Expression levels of *HTT* and *TTC19* in DN-Datasets. **(G)** Expression levels of *HTT* and *TTC19* in SP-Datasets. DN, Diabetic nephropathy; SP, Sarcopenia. The symbol ** is equivalent to P < 0.01, which is highly statistically significant. The symbol *** is equivalent to P < 0.001 and highly statistically significant. The closer the AUC in the ROC curve is to 1, the better the diagnostic effect is. When AUC was 0.7–0.9, it had a certain accuracy. AUC > 0.9 had high accuracy. ROC, Receiver operating characteristic curve; AUC, Area under the curve.

Using the LASSO algorithm, we identified 8 and 9 mitochondrial-related genes in the DN and SP datasets, respectively (Figures 4A–D). Using the RF approach, we pinpointed the top five genes in the DN and SP categories (Figures 4E–H), while the SVM algorithm identified 8 and 10 genes in DN and SP, respectively (Figures 5A–D), and the XGB algorithm identified 7 genes in both DN and SP (Figures 5E,F). By taking the intersection of the results from these four algorithms, we identified two key mitochondrial-related genes (*HTT* and *TTC19*) (Figures 6A–C). We then used ROC curves to estimate the diagnostic value of these two key genes (Figures 6D,E); it was observed that *HTT* was upregulated in DN (Figure 6F), while *TTC19* was upregulated in DN and downregulated in SP (Figure 6G).

### 3.4 Construction and evaluation of the diagnostic model

**FIGURE 7:**
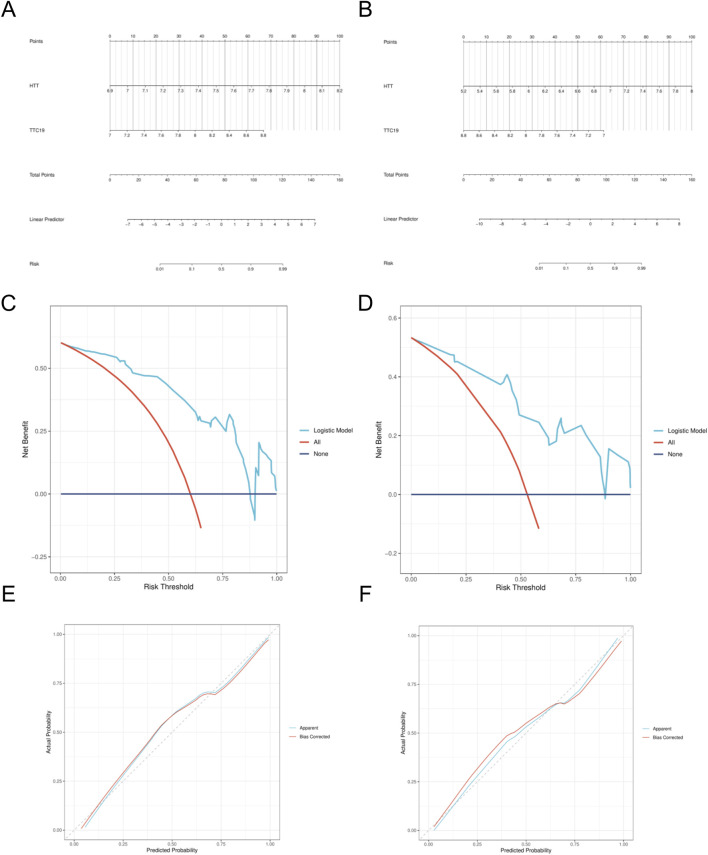
Construction of nomogram model. **(A)** Nomogram predicting the probability of DN. **(B)** Nomogram predicting the probability of SP. **(C)** DCA curves of the DN risk models. **(D)** DCA curves of the DN risk models. **(E)** Calibration curves of the DN risk models. **(F)** Calibration curves of the SP risk models.

A nomogram model was developed, incorporating the scores from the two hub genes. (Figures 7A,B). The decision curve analysis (DCA) curve showed that patients using this model benefited more than those without intervention or with full intervention (Figures 7C,D). The bias-adjusted curve closely approximated the perfect calibration curve, suggesting that the model was effectively calibrated (Figures 7E,F).

### 3.5 Immune infiltration analysis

**FIGURE 8:**
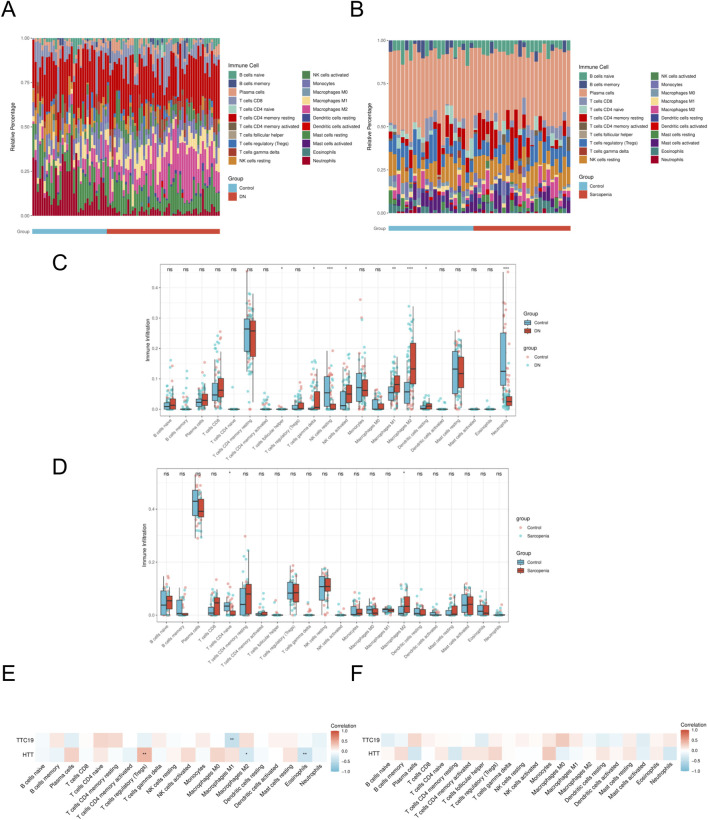
Immune cell infiltration analyses. **(A)** Immune cell distribution map in DN. **(B)** Immune cell distribution map in SP. **(C)** boxplot showing the comparison of 22 kinds of immune cells between DN and the control group. **(D)** boxplot showing the comparison of 22 kinds of immune cells between SP and the control group. **(E)** heatmap representing the associations of the differentially infiltrated immune cells with the two hub genes in DN. **(F)** heatmap representing the associations of the differentially infiltrated immune cells with the two hub genes in SP. DN, Diabetic nephropathy; SP, Sarcopenia. The symbol ** is equivalent to P < 0.01, which is highly statistically significant. The symbol *** is equivalent to P < 0.001 and highly statistically significant.

Utilizing the CIBERSORT method, we assessed the infiltration of immune cells in the case and control cohorts within the DN and SP dataset. Significant variations in the distribution of diverse immune cell populations were observed between DN and SP (Figures 8A,B). The DN group exhibited a significant increase in the number of M1 macrophages and M2 macrophages (Figure 8C), while SP patients demonstrated a significant elevation in the number of M2 macrophages (Figure 8D). Two pivotal genes were also found to be closely associated with multiple immune cells (Figures 8E,F). In the context of DN, *HTT* was positively correlated with regulatory T cells and negatively correlated with M2 macrophages and eosinophils, whereas *TTC19* was negatively correlated with M1 macrophages. The results imply that these genes could be critical in shaping the molecular and immune cell recruitment landscape in individuals with DN and SP.

### 3.6 Enrichment analysis of characteristic genes

**FIGURE 9:**
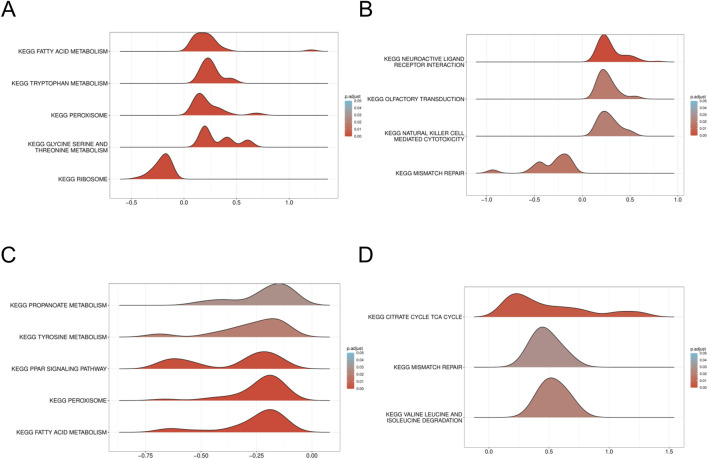
GSEA of DN Datasets dataset. **(A)** GSEA for the *HTT* in DN. **(B)** GSEA for the *HTT* in DN. **(C)** GSEA for the *TTC19* in SP. **(D)** GSEA for the *TTC19* in SP. GSEA, Gene set enrichment analysis; DN, Diabetic nephropathy; SP, Sarcopenia. The screening criteria of gene set enrichment analysis (GSEA) were p. Adj<0.05 and FDR value (q value) <0.25.

To gain a deeper understanding of the enrichment pathways associated with characteristic genes, we performed Gene Set Enrichment Analysis (GSEA) on a single-gene basis. Utilizing variations in expression levels, the target genes were sorted, thereby establishing groups characterized by high and low expression. The enrichment degree was evaluated by calculating the cumulative scores of the target gene sets in the ranked list. Enrichment analysis of single genes reveals that *HTT* and *TTC19* are significantly associated with mitochondrial and fatty acid metabolism in patients with DN (Figures 9A,B), while in patients with SP, *HTT* and *TTC19* were found to be enriched within the mismatch repair signaling pathway (Figures 9C,D).

### 3.7 Drug prediction and molecular docking

**FIGURE 10:**
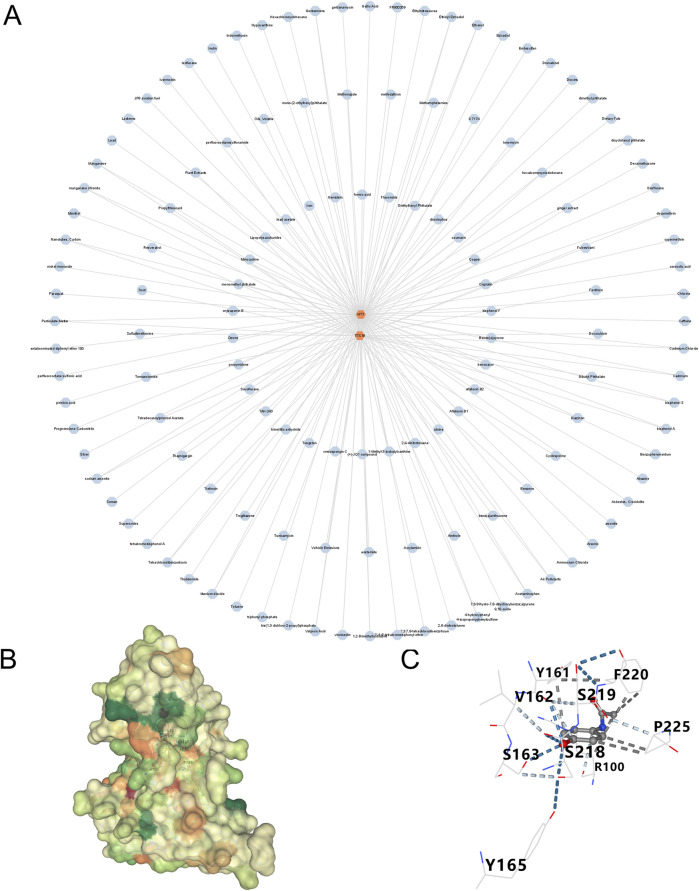
Prediction of drugs for three genes **(A)** A drug-genes network. **(B,C)** Schematic diagram of resveratrol-*HTT* molecular docking.

Utilizing the Cytoscape, we developed a network mapping drugs to genes (Table 2). The results indicated that acetaminophen targets two genes (Figure 10A). Subsequently, we employed molecular docking techniques to evaluate the binding stability with the two hub genes in order to identify the optimal gene. Typically, a binding energy below −5 kcal/mol is considered to denote a high binding affinity, and a score lower than −7 kcal/mol is indicative of a strong binding interaction. A strong binding interaction is observed between acetaminophen and HTT, characterized by a binding energy of −6.2 kcal/mol (Figures 10B,C).

**TABLE 2:** mRNA-Drug interaction network nodes.

mRNA	Drug
HTT	Cadmium Chloride
HTT	Bisphenol A
HTT	Manganese
HTT	Minocycline
HTT	Aflatoxin B1
HTT	Tetrachlorodibenzodioxin
HTT	Vinclozolin
HTT	Atrazine
HTT	Bisphenol F
HTT	Cadmium
HTT	Copper
HTT	Dioxins
HTT	Dronabinol
HTT	Endosulfan
HTT	Ethinyl Estradiol
HTT	Indomethacin
HTT	Isoflurane
HTT	Lead
HTT	Lipopolysaccharides
HTT	Manganese chloride
HTT	Nanotubes, Carbon
HTT	1,2-Dimethylhydrazine
HTT	1-Methyl-3-isobutylxanthine
HTT	2,2′,4,4′-tetrabromodiphenyl ether
HTT	2,3,7,8-tetrachlorodibenzofuran
HTT	2,4-dinitrotoluene
HTT	2,6-dinitrotoluene
HTT	4-hydroxyphenyl 4-isopropoxyphenylsulfone
HTT	7,8-Dihydro-7,8-dihydroxybenzo(a)pyrene 9,10-oxide
HTT	Abrine
HTT	Acetaminophen
HTT	Aflatoxin B2
HTT	Air Pollutants
HTT	Ammonium Chloride
HTT	Arsenic
HTT	Arsenite
HTT	Asbestos, Crocidolite
HTT	Benoxacor
HTT	Benzo(a)pyrene
HTT	Benzophenoneidum
HTT	Bisphenol S
HTT	Caffeine
HTT	Chlorine
HTT	Cisplatin
HTT	Corosolic acid
HTT	Coumarin
HTT	Cypermethrin
HTT	Decamethrin
HTT	Desflurane
HTT	Dexamethasone
HTT	Dicrotophos
HTT	Dicyclohexyl phthalate
HTT	Dietary Fats
HTT	Diethylhexyl Phthalate
HTT	Dimethyl phthalate
HTT	Estradiol
HTT	Ethanol
HTT	Ethylnitrosourea
HTT	Flavonoids
HTT	Formic acid
HTT	FR900359
HTT	Gallic Acid
HTT	Geldanamycin
HTT	Genistein
HTT	Gentamicins
HTT	Hexachlorocyclohexane
HTT	Hypoxanthine
HTT	Inulin
HTT	Iron
HTT	Ivermectin
HTT	JP8 aviation fuel
HTT	(+)-JQ1 compound
HTT	Lactones
HTT	Lead acetate
HTT	Menthol
HTT	Monomethyl phthalate
HTT	Nickel monoxide
HTT	Onjisaponin B
HTT	Ozone
HTT	Paraquat
HTT	Particulate Matter
HTT	Pentabrominated diphenyl ether 100
HTT	Perfluorooctane sulfonic acid
HTT	Pirinixic acid
HTT	Pregnenolone Carbonitrile
HTT	Procymidone
HTT	Sevoflurane
HTT	Silver
HTT	Sodium arsenite
HTT	Soman
HTT	Superoxides
HTT	TAK-243
HTT	Tetrabromobisphenol A
HTT	Thalidomide
HTT	Titanium dioxide
HTT	Toluene
HTT	Trimellitic anhydride
HTT	Triphenyl phosphate
HTT	Tris (1,3-dichloro-2-propyl)phosphate
HTT	Tungsten
HTT	Valproic Acid
HTT	Xestospongin C
TTC19	Valproic Acid
TTC19	Bisphenol A
TTC19	Acetaminophen
TTC19	Cadmium
TTC19	Dibutyl Phthalate
TTC19	Nanotubes, Carbon
TTC19	Tetrachlorodibenzodioxin
TTC19	Vehicle Emissions
TTC19	1,2-Dimethylhydrazine
TTC19	4-hydroxyphenyl 4-isopropoxyphenylsulfone
TTC19	Acetamide
TTC19	Acrylamide
TTC19	Air Pollutants
TTC19	Amitrole
TTC19	Benz(a)anthracene
TTC19	Benzene
TTC19	Bisphenol S
TTC19	Cadmium Chloride
TTC19	Cyclosporine
TTC19	Decamethrin
TTC19	Diazinon
TTC19	Doxorubicin
TTC19	Ethanol
TTC19	Ethinyl Estradiol
TTC19	Fenthion
TTC19	Fulvestrant
TTC19	Gentamicins
TTC19	Ginger extract
TTC19	Hexabromocyclododecane
TTC19	Ionomycin
TTC19	K 7174
TTC19	Manganese
TTC19	Manganese chloride
TTC19	Methamphetamine
TTC19	Methidathion
TTC19	Methimazole
TTC19	mono-(2-ethylhexyl)phthalate
TTC19	Oils, Volatile
TTC19	Particulate Matter
TTC19	Perfluorooctanesulfonamide
TTC19	Plant Extracts
TTC19	Propylthiouracil
TTC19	Resveratrol
TTC19	Sodium arsenite
TTC19	Soot
TTC19	Sulfadimethoxine
TTC19	Temozolomide
TTC19	Tetradecanoylphorbol Acetate
TTC19	Thapsigargin
TTC19	Titanium dioxide
TTC19	Tretinoin
TTC19	Troglitazone
TTC19	Tunicamycin
TTC19	Vinclozolin

## 4 Discussion

Diabetic nephropathy (DN), a prevalent complication of diabetes mellitus, has emerged as a leading global contributor to end-stage renal failure. Identifying reliable biomarkers for chronic kidney disease (CKD), especially DN, is crucial for early detection and understanding the disease. Recent advancements have introduced a variety of novel biomarkers, including protein-based biomarkers such as serum secreted leukocyte protease inhibitor (SLPI), which has proven to be a valuable predictor of DN progression (Sun et al., 2024). Urine biomarkers, such as neutrophil gelatinase-associated lipocalin (NGAL), kidney injury molecule-1 (KIM-1), and periostin, have shown potential as tools for the noninvasive monitoring of DN (V et al., 2024). Microbiome interventions, such as targeting *Lactobacillus* johnsonii, have been proposed as potential strategies for reversing CKD (Miao et al., 2024a). Metabolite-based biomarkers, including p-cresol sulfate and indole sulfate, have been investigated for their role in predicting renal function based on glomerular filtration rate (GFR) categories (Corradi et al., 2024). Additionally, research on the diagnostic complexities of CKD in the elderly has highlighted the importance of considering biomarkers specifically for this population (Muglia et al., 2024). Furthermore, metabolites such as serum 5-methoxytryptophan (5-MTP), homocysteine, and citrulline have been identified as biomarkers in patients with advanced DN through a combination of untargeted and targeted metabolomics approaches (Chen et al., 2024). A hyperglycemic environment fosters the overproduction of reactive oxygen species (ROS), leading to mitochondrial DNA (mtDNA) impairment and ultimately resulting in renal impairment (Takasu et al., 2025). Emerging therapeutic strategies targeting mitochondrial homeostasis, particularly through AMP-activated protein kinase (AMPK) activation and nitric oxide (NO) production modulation, demonstrate potential for halting DN progression (Börgeson et al., 2017; Gao et al., 2004). Concurrently, mitochondrial DNA content serves as a critical biomarker for muscle injury and exhibits strong associations with sarcopenia (SP) progression (Short et al., 2005; Rygiel et al., 2016). Given the high mitochondrial density in skeletal muscle fibers essential for ATP production via oxidative phosphorylation, mitochondrial dysfunction has been implicated as a central mechanism in SP pathogenesis (Calvani et al., 2013). Through systematic cross-disease analysis, we identified mitochondrial-related hub genes shared between DN and SP, revealing their pivotal role in disease pathogenesis.

In this study, we identified 80 genes exhibiting similar patterns in two diseases, including 10 genes related to mitochondrial function: *CKB*, *FAM162A*, *HSPE1*, *HTT*, *NFKBIA*, *PPARGC1A*, *PRKDC*, *QDPR*, *TTC19*, and *UCHL1*. Machine learning algorithms prioritized *HTT* and *TTC19* as core diagnostic biomarkers, with logistic regression models demonstrating high discriminative accuracy. Gene Ontology (GO) and Kyoto Encyclopedia of Genes and Genomes (KEGG) enrichment analyses indicated that these genes are significantly involved in the regulation of circadian rhythms, cellular metabolism, and apoptosis. These findings suggest two important considerations: first, the regulation of circadian rhythms, cellular metabolism, and apoptosis may constitute a shared pathogenic mechanism in both diseases; second, impaired mitochondrial function could potentially influence these processes.

*HTT* (Huntingtin) is a multifunctional cytoplasmic protein (Prowse et al., 2025), whose expression is downregulated in both DN and SP as demonstrated in our study. *HTT* interacts with proteins such as dynein to regulate vesicular transport and synaptic transmission, which are essential for maintaining the normal physiological functions of neurons and muscle cells (Prowse et al., 2025). Furthermore, *HTT* plays a critical role in the regulation of mitochondrial function, impacting adenosine triphosphate (ATP) production and oxidative stress (Prowse et al., 2025; Browne et al., 1999). In the brains of patients with Huntington’s disease (HD), markers of oxidative stress, including 8-hydroxy-2′-deoxyguanosine and malondialdehyde (MDA), are significantly elevated (Polidori et al., 1999). Observations have indicated the presence of oxidative stress in the peripheral blood of individuals with HD (Chen et al., 2007), as well as in animal models simulating HD (Bogdanov et al., 2001). Oxidative stress plays a pivotal role in the onset of DN (Samsu, 2021; Jin et al., 2023) and is a major pathogenic factor in SP (Chen et al., 2022), characterized by neurogenic denervation at the neuromuscular junction, muscle fiber atrophy, and mitochondrial dysfunction (Xu et al., 2025). We propose that *HTT* contributes to mitochondrial damage in patients with DN and facilitates the progression of SP. Tetratricopeptide repeat domain 19 (*TTC19*) encodes a protein with a molecular weight of 35 kDa, which was initially considered a complex III (CIII) assembly factor. This discovery was based on the detection of the absence of this protein in some biochemical patients presenting with isolated CIII deficiency (Ghezzi et al., 2011). Through in - depth molecular studies on the Ttc19^−/−^ mouse model, we found that *TTC19* plays an important role in clearing the N - terminal fragments generated during the processing of the catalytic Fe - S Rieske subunit (Uqcrfs1), which is crucial for maintaining the functional and structural integrity of CIII (Bottani et al., 2017; Fernandez-Vizarra and Zeviani, 2018). This complex is essential for mitochondrial function, and any disruptions in its function can result in mitochondrial dysfunction. *TTC19* is integral to mitochondrial dynamics; however, research examining the association between *TTC19* and DN remains limited. Despite this, *TTC19* expression is linked to mitochondrial dysfunction, a process critically involved in the development and progression of both DN (Zhang et al., 2021) and SP (Kamarulzaman and Makpol, 2025). Therefore, addressing mitochondrial dysfunction is a fundamental concern in the management of DN and SP.

Gene Set Enrichment Analysis (GSEA) reveals that *HTT* and *TTC19* are significantly enriched in the mitochondrial and fatty acid metabolism pathways in individuals with DN, whereas these genes are overrepresented in the mismatch repair signaling pathway among individuals with SP. As mentioned above, the functional abnormalities of *HTT* and *TTC19* can lead to mitochondrial dysfunction. The enrichment of *HTT* and *TTC19* in mitochondrial and fatty acid metabolic pathways in patients with DN may suggest a correlation between the functional anomalies of these genes and the mitochondrial dysfunction and fatty acid metabolic dysregulation observed in DN. In DN, the enrichment of mitochondrial and fatty acid metabolism pathways suggests a disruption in energy homeostasis and an increase in oxidative stress. This mitochondrial dysfunction likely contributes to key pathological processes, including epithelial-mesenchymal transition (EMT), which is a well-established driver of renal fibrosis in DN (Balakumar, 2024). Moreover, mitochondrial impairment is critically associated with podocyte injury, another hallmark of DN. Podocytes, which are essential for maintaining the integrity of the glomerular filtration barrier, are particularly vulnerable to mitochondrial damage caused by hyperglycemia and oxidative stress (Liu et al., 2022). Protective mechanisms, such as the upregulation of Sirtuin 6 (Sirt6), have been demonstrated to alleviate podocyte injury by inhibiting harmful pathways, including the Wnt/β-catenin signaling pathway and the renin-angiotensin system (RAS) (Miao et al., 2024b). The interaction between mitochondrial health and these crucial signaling pathways, which are dysregulated in DN, such as RAS and Wnt/β-catenin, highlights the fundamental importance of maintaining mitochondrial homeostasis (Balakumar, 2024; Miao et al., 2024b). The observed enrichment of *HTT* and *TTC19* in the mismatch repair signaling pathway among SP patients suggests a potential pivotal role for these genes in the maintenance of genomic stability. Mismatch repair constitutes a fundamental component of the cellular DNA damage repair system, crucial for averting the accumulation of mutations and preventing genomic instability. The presence of *HTT* and *TTC19* enrichment within this pathway may indicate compromised DNA repair capabilities in SP patients (Narciso et al., 2007). Furthermore, the variation in enrichment pathways of *HTT* and *TTC19* across different patient populations suggests that these genes may perform distinct functional roles under varying pathological conditions. This observation carries significant implications for elucidating the molecular mechanisms underlying DN and SP.

Our study found a significant increase in macrophages in DN, consistent with their known role as the main immune cells in renal biopsies for DN (Tesch, 2010; Liu et al., 2021). In addition to macrophages, the dysregulation of other immune cell populations plays a substantial role in the pathogenesis of DN. For example, emerging subsets of T-cells have been identified as non-invasive biomarkers for vascular injury during the pre-dialysis stages of CKD (Martín-Vírgala et al., 2024), underscoring the extensive involvement of adaptive immunity. The activation of macrophages and the progression of DN are driven by factors such as advanced glycation end-products (AGEs), oxidized low-density lipoprotein (Ox-LDL), reactive oxygen species (ROS), and proteases (Hickey and Martin, 2013). These mechanisms exacerbate tissue damage, ultimately leading to renal fibrosis. Moreover, renal injury is notably prevalent among patients with idiopathic inflammatory myopathies (IIMs) (Conticini et al., 2023). Biomarkers such as neutrophil gelatinase-associated lipocalin (NGAL), kidney injury molecule-1 (KIM-1), Activin A, CD163, and Cystatin C have proven to be reliable for the early diagnosis of CKD in this context (Conticini et al., 2023), thereby reinforcing the crucial connection between immune dysregulation, and renal damage across different conditions. In addition, clinical studies have demonstrated age-related variations and changes in macrophage phenotype responses in human skeletal muscle following injury (Zhang et al., 2024). Muscle biopsies from older adults (aged 60–75 years) exhibit a significant decrease in CD68+CD11b+M1 macrophages compared to younger individuals (aged 18–35 years). Furthermore, an 8-week lower limb eccentric exercise rehabilitation program resulted in a more pronounced increase in CD206+M2 macrophages in older adults than in younger individuals (Reidy et al., 2018), aligning with our research findings.

A variety of chemical compounds and medications have been explored as potential treatments for DN and SP. Nonetheless, as of now, no definitive pharmacological interventions have been pinpointed for patients suffering from both DN and SP. Our research aimed to identify potential therapeutic targets and found acetaminophen to be a promising treatment option for both conditions. While predicting acetaminophen-*HTT* binding computationally is intriguing, clinical validation of *HTT* is crucial. Specific miRNAs in urine exosomes, like miR-320a, are early biomarkers for kidney disease (Mishra et al., 2024). Exosome-based therapies, such as delivering miRNA-23a/27a/26a clusters, have shown promise in reducing tubulointerstitial fibrosis in DN models (Ji et al., 2023). Additionally, using specific *Lactobacillus* strains, such as *Lactobacillus* reuteri, can improve membranous nephropathy by affecting the aryl hydrocarbon receptor pathway (Miao et al., 2024c). These findings emphasize the potential of targeting the gut-kidney axis and microbial metabolites. Consequently, future research should concentrate on two main areas: validating the diagnostic and prognostic value of miRNA biomarkers through non-invasive methods like urinary exosomal analysis, and advancing clinical research on new therapies, including exosomes and microbial interventions, to evaluate their effectiveness and safety in patients.

Although prior research has noted links between DN and SP, the common molecular pathways underlying these diseases have not been analyzed through bioinformatics methods. Our study marks the first identification of key mitochondrial genes that may be implicated in the pathogenesis of DN and SP. Through the application of sophisticated machine learning techniques, we have illuminated potential connections between these genes and the overlapping pathological mechanisms of DN and SP. Nevertheless, our study has certain limitations. Firstly, the study’s dataset, sourced from public repositories, had limited and uneven data, potentially missing key genes in disease progression. Secondly, although the predictive model was developed and evaluated using cross-validation with strict data partitioning to reduce overfitting risks, external validation in independent clinical cohorts remains necessary. Thirdly, the four models built on small public datasets may have limited generalizability despite cross-validation. Future studies should expand cohorts and employ algorithms tailored for small samples (e.g., sPLS-DA) to enhance reliability. Fourthly, the current overlap of the results of the four machine learning models relies on empirical intersections, and in the future, consensus strategies such as weighted voting and integrated learning need to be introduced and combined with statistical tests (e.g., Bootstrapping) to quantify gene selection stability.

Ultimately, to corroborate our findings, the pathological causal pathways of diseases linked to key genes and immune infiltration require additional experimental confirmation from external sources. This study investigated the relationship between DN and SP through transcriptomic data analysis, identifying common DEGs and hub genes pertinent to both conditions. Multiple bioinformatics analyses were conducted based on these findings. The research indicates that DN and SP share certain pathogenic mechanisms potentially mediated by specific key genes. The present research provides innovative biological targets and understanding, which can enhance investigations into the molecular pathways, facilitate the creation of novel treatments, and support the early detection and efficient management of individuals suffering from DN and SP. Identifying *HTT* and *TTC19* as crucial links between mitochondrial dysfunction and DN pathogenesis highlights their potential as therapeutic targets. These genes are enriched in pathways vital for DN, such as mitochondrial and fatty acid metabolism, which may affect EMT and podocyte injury. Targeting these genes or their downstream effects, like modulating mitochondrial function or protecting podocytes, shows promise. Innovative approaches, such as exosome-mediated miRNA delivery or microbiome modulation, expand the therapeutic options for DN. However, the biological implications of these findings necessitate additional investigation, which should be conducted through a combination of *in vitro* and *in vivo* experimental approaches.

## 5 Conclusion

We identified two mitochondrial-related hub genes that are common to both DN and SP, demonstrating their significant diagnostic value. The genes *HTT* and *TTC19* are implicated in the mitochondrial metabolic pathways of these conditions. Our investigation reveals new understanding of the common pathological processes involved in DN and SP. However, further clinical research is essential to confirm the functions and pathways of these genes in individuals affected by DN and SP.

## Data Availability

The original contributions presented in the study are included in the article/Supplementary Material, further inquiries can be directed to the corresponding authors.
