# Supplementary figures and images for: Mitochondrial insights: key biomarkers and potential treatments for diabetic nephropathy and sarcopenia
Source: Front Cell Dev Biol. 2025 Jul 9;13:1596204. doi: 10.3389/fcell.2025.1596204 (PMC12283731; doi:10.3389/fcell.2025.1596204)

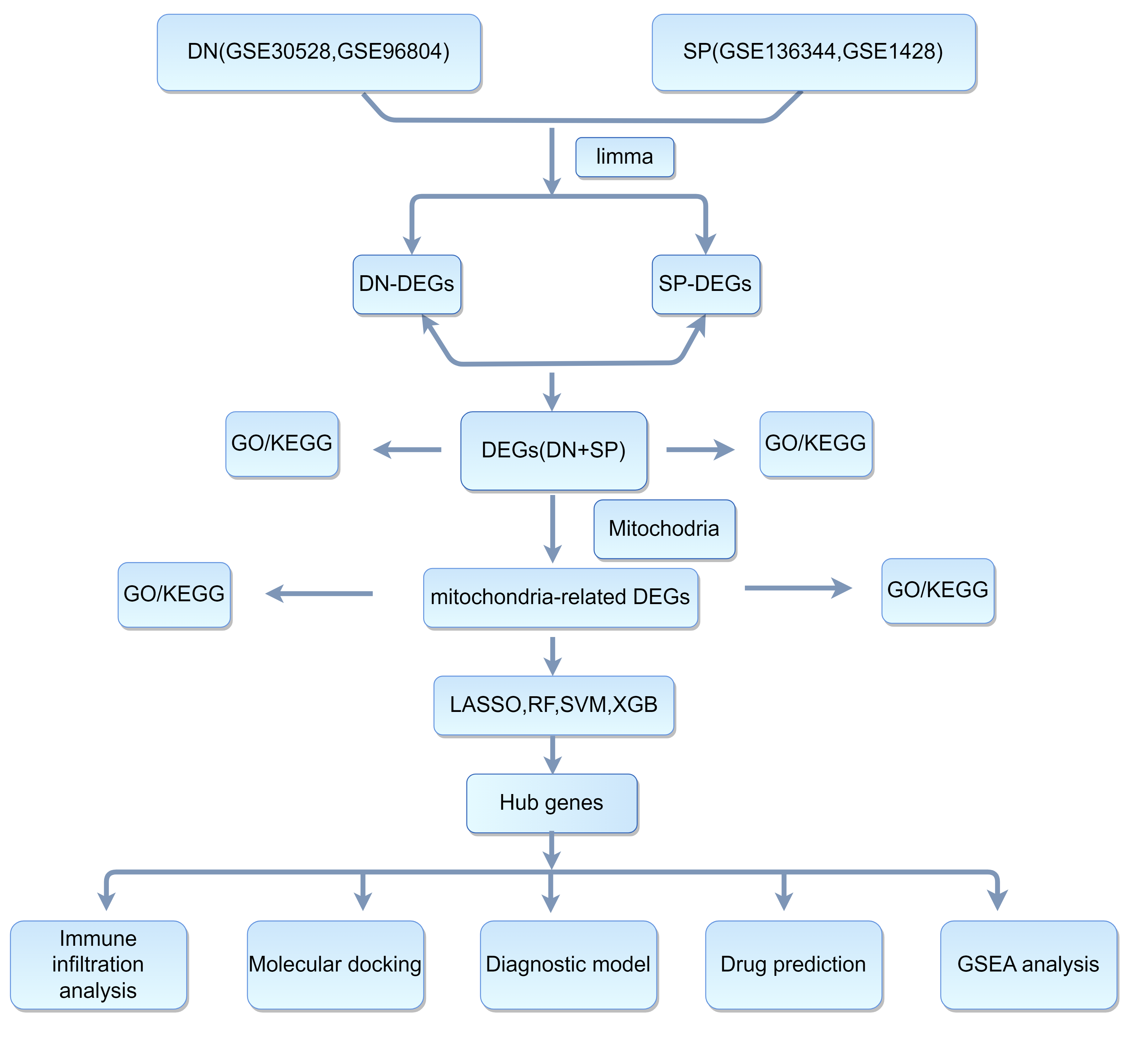

Supplement: Supplementary file 1 [file Image1.tif]
